# Donor-specific toxicity of copanlisib identified using immune-humanized mice

**DOI:** 10.1007/s00262-026-04398-9

**Published:** 2026-06-12

**Authors:** Robyn E. Becker, Jenny Résiliac, Kathleen L. Gabrielson, Zhuoming Liu, Kristina E. Howard

**Affiliations:** 1 Division of Applied Regulatory Science, OCP/OTS/CDER, U.S. Food & Drug Administration, 10903 New Hampshire Ave, Silver Spring, MD 20993 USA; 2 Department of Molecular and Comparative Pathobiology, Johns Hopkins University, 733 North Broadway, Miller Research Building, Room 807, Baltimore, MD 21205-2196 USA; 3 Present Address: Cellular and Tissue Therapy Branch, DCT1/OCTHT/OTP/CBER, U.S. Food & Drug Administration, 10903 New Hampshire Ave, Silver Spring, MD USA

**Keywords:** Copanlisib, Toxicity, Immune-humanized mice, Inflammation

## Abstract

Copanlisib is a pan-phosphoinositide-3-kinase-inhibitor originally indicated for adult patients with relapsed follicular lymphoma who had received at least two prior systemic therapies. At the time this study was conducted, it was approved for use by the FDA and was being evaluated in clinical trials in combination therapy with checkpoint inhibitors for a range of cancers.

Prior to conducting studies with the combination of copanlisib and checkpoint inhibitors, we conducted a pilot study to determine route of administration, identify an appropriate dose, and assess the effects of copanlisib in bone marrow-liver-thymus (BLT) immune-humanized mice. We tested three doses of copanlisib via two routes of administration in humanized mice produced from three unique donors and evaluated tissue-based toxicity via histopathology and immune changes with flow cytometry.

Histopathology showed no significant correlation in pathological findings based on copanlisib dose or administration route. However, analysis of the histopathology by donor revealed clear differences, with one donor showing significant pathology, regardless of treatment dose or route, while the other two donors showed little to no adverse treatment effects. Flow cytometric analysis showed the affected donor had significantly more activated T-cells present in the bone marrow regardless of dose or route of administration.

Although our findings represent only one of three donors used in immune-humanized mice, this study suggests that some immune modulating therapeutic toxicity could be associated with specific genetic factors from individual patients rather than being dose related.

**Supplementary Information:**

The online version contains supplementary material available at 10.1007/s00262-026-04398-9.

## Introduction

Over the past decade, copanlisib was considered a preferred pan-phosphoinositide-3-kinase (PI3k) inhibitor for use in combination therapy treatment, or as a second- or third-line treatment, with single-arm clinical trials resulting in positive outcomes [1, 2]. However, not all clinical trials have had positive results, with several halted early due to treatment-emergent adverse events (TEAEs) [3, 4], and some showing no clinical benefit with increased TEAEs [5]. Most clinical trials studying PI3k inhibitors have reported varying levels of TEAEs including dermatitis, autoimmune dysfunction, hyperglycemia, and hypertension [6]. Copanlisib was withdrawn from the market in March 2024 after the CHRONOS-4 study failed to provide conclusive evidence of clinical benefits in combination therapy, while demonstrating higher levels of TEAE’s than in the previous CHRONOS-3 study [7].

To better understand TEAEs arising from combination therapies, we utilized immune-humanized mice as an animal model. They are engineered to have a human immune system engrafted into a severely immunocompromised mouse strain [8]. This model allows for the testing of biological drug products that specifically engage human immune receptors. It also permits study of drug product effects on the human immune system. In our previous studies, we demonstrated that these mice can exhibit immune-related adverse effects (irAEs) such as cytokine release syndrome [9, 10] and irAEs similar to those observed with checkpoint inhibitors such as nivolumab [11].

This report presents the results of a pilot study evaluating treatment-related adverse effects TRAEs from copanlisib in bone marrow-liver-thymus (BLT) immune-humanized mice. Our goals were to establish a dosing range, route of administration, and to determine if, and in what dose range, they developed adverse events. Data derived from the pilot study were then used to conduct combination therapy studies that included copanlisib and checkpoint inhibitor (CI) products.

## Materials and methods

### Reagents

Copanlisib (BAY 80–6946) was a generous gift from NCI-Chemotherapeutic Agents Repository (Germantown, MD). Sterile saline was purchased from Med-Vet International (Mettawa, IL). Flow cytometry antibodies were purchased from BioLegend, BD Biosciences, and eBioscience.

### Mice with humanized immune systems

All animal procedures were approved by the Institutional Animal Care and Use Committee, Center for Drug Evaluation and Research, FDA, and were carried out in an AAALAC-accredited facility. Female NOG-IL6 mice were purchased from Taconic Biosciences (Germantown, NY). All housing and husbandry conditions, as well as surgery to humanize the mice, were as previously described [11].

### Experimental design

Mice were generated using tissue and stem cells from three unique donors (Table 1). It was unknown how well copanlisib would be tolerated; and given the reports of neurologic issues with IV dosing in conventional mice, two different routes of administration (with different dosing schedules) were selected based on prior conventional mouse studies [12, 13]. One–two humanized mice from each donor were randomly assigned to each treatment group (saline IP, 4 mg/kg IP or IV, 8 mg/kg IP or IV, 16 mg/kg IP or IV) yielding seven total treatment groups with *n* = 4, for a total of 28 mice. Details are shown by treatment and donor in Supplemental Tables1, 2, 3. The IV groups received a once-weekly dosing for three weeks, divided into two injections on the same day to minimize toxicity. The IP groups were dosed every other day for 11 intervals. Data from conventional mice enabled dose selection to ensure that, if possible, adverse events would occur [12, 13].

**Table 1:** Class I and class II HLA alleles by donors

	HLA-A	HLA-B	HLA-C	HLA-DPB1	HLA-DQB1	HLA-DRB1
Donor A	24:02	–:–	04:01	04:01	05:02	15:01
	74:01	–:–	07:02	61:01	06:02	11:04
Donor B	23:01	13:02	03:04	02:01	03:01	11:04
	24:02	40:01	06:02	14:01	06:04	13:02
Donor C	02:01	27:05	02:02	04:01	05:01	01:01
	02:01	40:01	03:04	04:01	06:04	13:02

### Necropsy, histopathology, and flow cytometry

Mice were killed between 21 and 24 days following their first dose. Tissues collected included heart, lung, liver, gastrointestinal tract, kidneys, bladder, spleen, sternum, femur, skin, brain, and endocrine organs. They were fixed in 10% formalin, processed into slides, and stained with hematoxylin and eosin (H&E) for pathology assessment by a board-certified pathologist who was blinded to donor and treatment status. Blood was collected and processed at day 0, 14 and necropsy. Spleen and bone marrow were harvested, processed into single-cell suspensions, stained, and analyzed by flow cytometry as previously described [11], with gating shown in Supplemental Fig. 1. Data were acquired using a Cytek Aurora 5L (Cytek Biosciences, Fremont, CA). Flow cytometry data analysis was performed using FCS Express. Statistics were calculated using GraphPad Prism.

## Results

### Histopathology

**Fig. 1:**
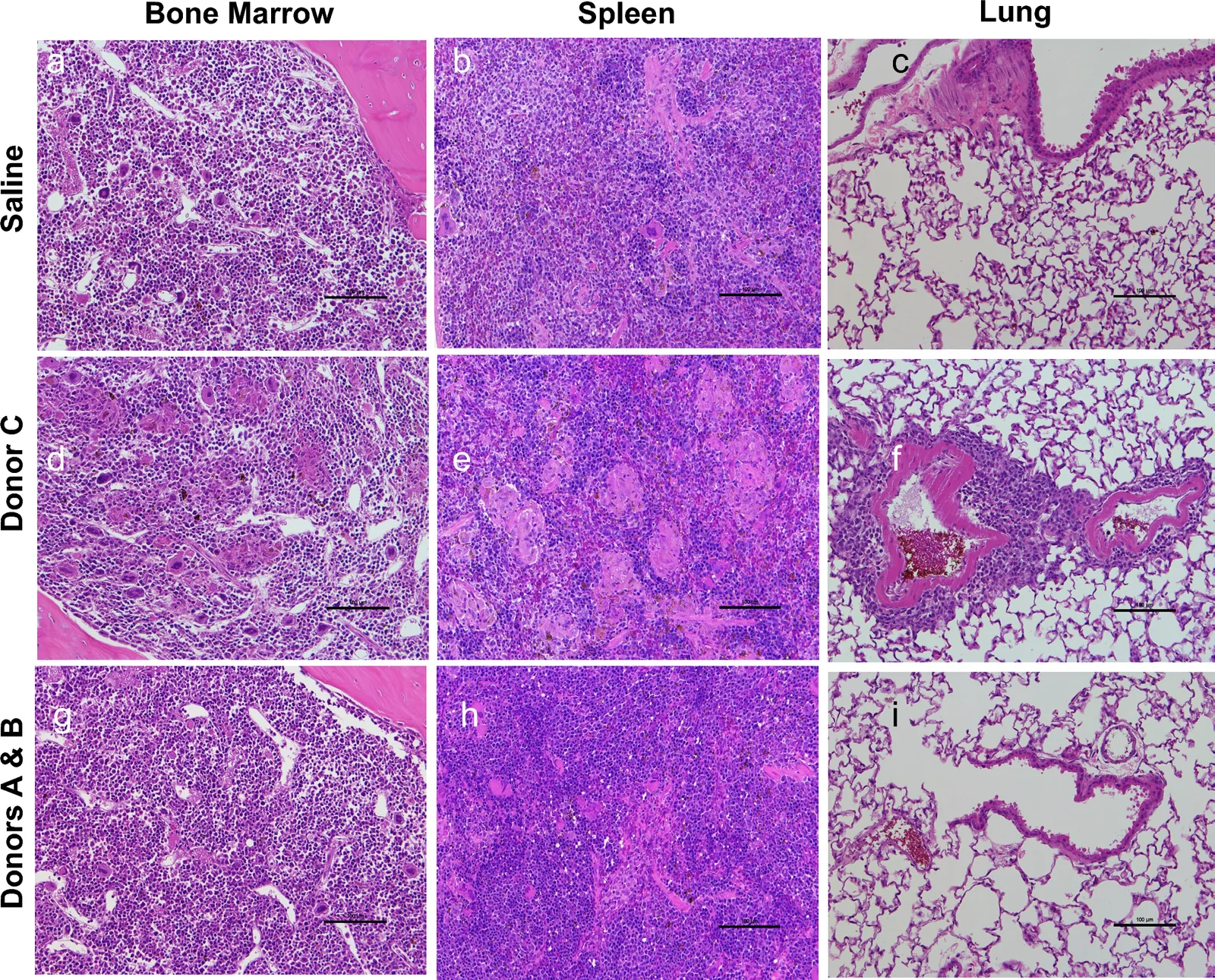
Bone marrow, spleen, and lung pathology. Representative tissues from saline (top row), donor C (middle row), and either donor A or B are shown (bottom row). Bone marrow sections (left panel) show little to no pathology for saline control mice (**a**) or donor A/B mice (**g**), while donor C mice (**d**) show mild, moderate to severe granulomas. Spleen sections (middle panel) show multiple granulomas in donor C (e) and extramedullary hematopoiesis observed in all mice (b, e, h), regardless of treatment, as it is commonly observed in humanized mouse spleens. Lung sections (right panel) show no pathology for saline control mice (**c**) or donor A/B mice (**i**), while donor C mice (**f**) show focal, mild lymphohistiocytic inflammation in tissues

**Fig. 2:**
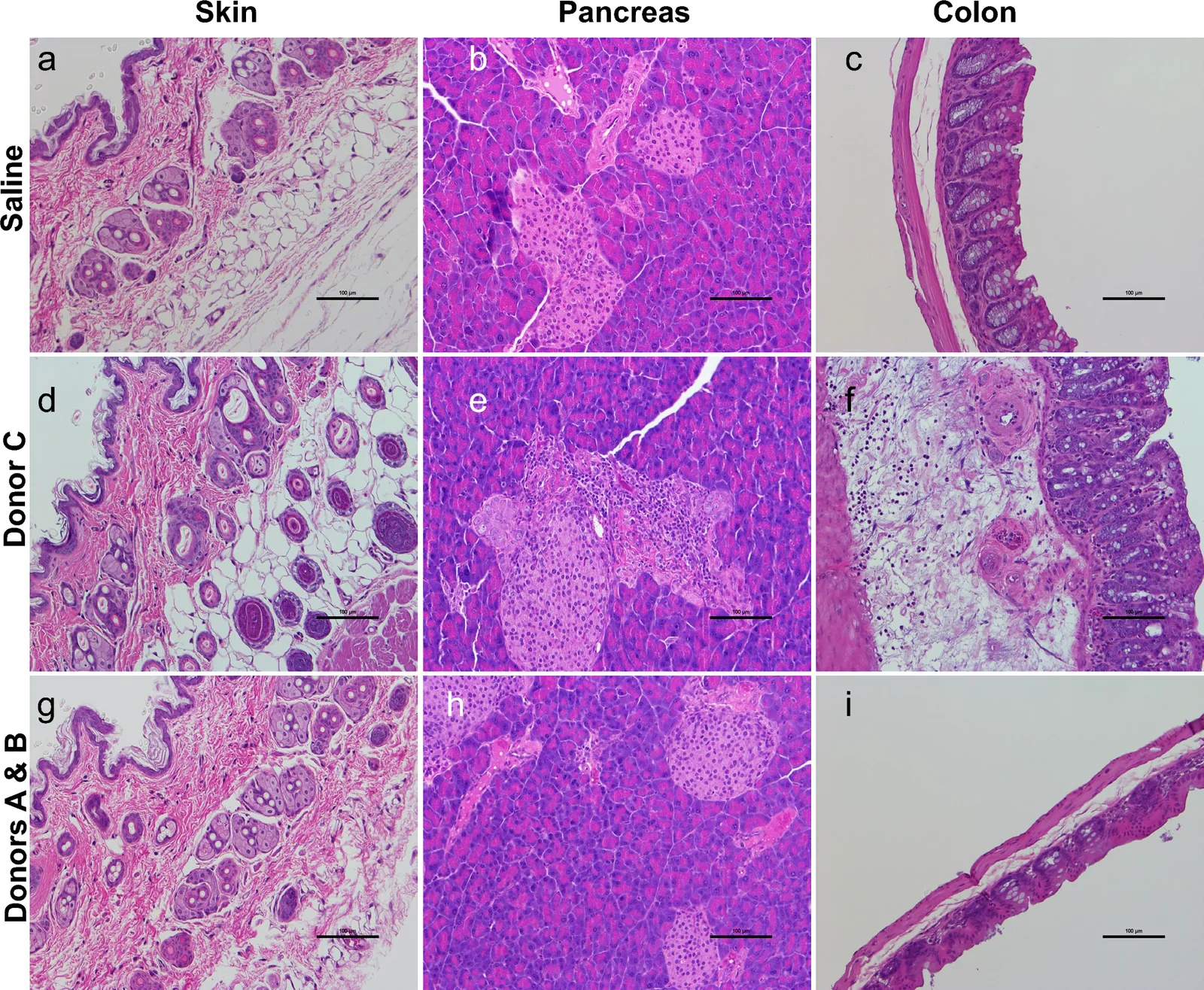
Skin, pancreas, and colon pathology. Representative tissues from saline (top row), donor C (middle row), and donor A or B (bottom row) are shown. Skin sections (left panel) show no pathology for saline control mice (**a**) or donor A/B mice (**g**), while donor C mice (**d**) show mild lymphohistiocytic inflammation. Pancreas sections (middle panel) show no pathology for saline control mice (**b**) or donor A/B mice (**h**), while donor C mice (**e**) show mild to moderate lymphohistiocytic inflammation. Colon sections (right panel) show no pathology for saline control mice (**c**) or donor A/B mice (**i**), while donor C mice (**f**) show varying degrees of inflammation, including severe edema and hemorrhage in tissues

Initially, histopathological analysis found no correlation between pathological findings and either the dose or administration route of copanlisib. However, when the data were analyzed by donor, clear differences emerged. Donor C exhibited significant pathological changes, regardless of treatment dose or route, while saline-treated and copanlisib treated donors A and B showed minimal to no adverse effects (Figs. 1, 2; Supplemental Table 4).

Specifically, bone marrow had mild to moderate numbers of granulomas (Fig. 1d); spleen contained multiple granulomas present throughout the tissue (Fig. 1e); and lung had focal, mild lymphohistiocytic inflammation, particularly around pulmonary arteries and veins in mice engrafted with the affected donor (Fig. 1f). Control (Fig. 1a–c) and unaffected donors (Fig. 1h–i) show no adverse pathology. Spleens of all humanized mice, whether control or treated, had varying degrees of mild to moderate extramedullary hematopoiesis present (Fig. 1b, e, g), which is commonly observed in this model (11).

Skin, pancreas, and colon show no adverse pathology for control (Fig. 2a–c) or donors A and B engrafted mice (Fig. 2g–i). In contrast, skin from mice engrafted with the affected donor (C) shows mild lymphohistiocytic inflammation in the dermis (Fig. 2d) and pancreas has focal areas of mild lymphohistiocytic inflammation (Fig. 2e). Colon for the affected donor displays a range of inflammation including severe edema, hemorrhage, and neutrophilic inflammation in all layers of colon (Fig. 2f).

### Flow cytometry

**Fig. 3:**
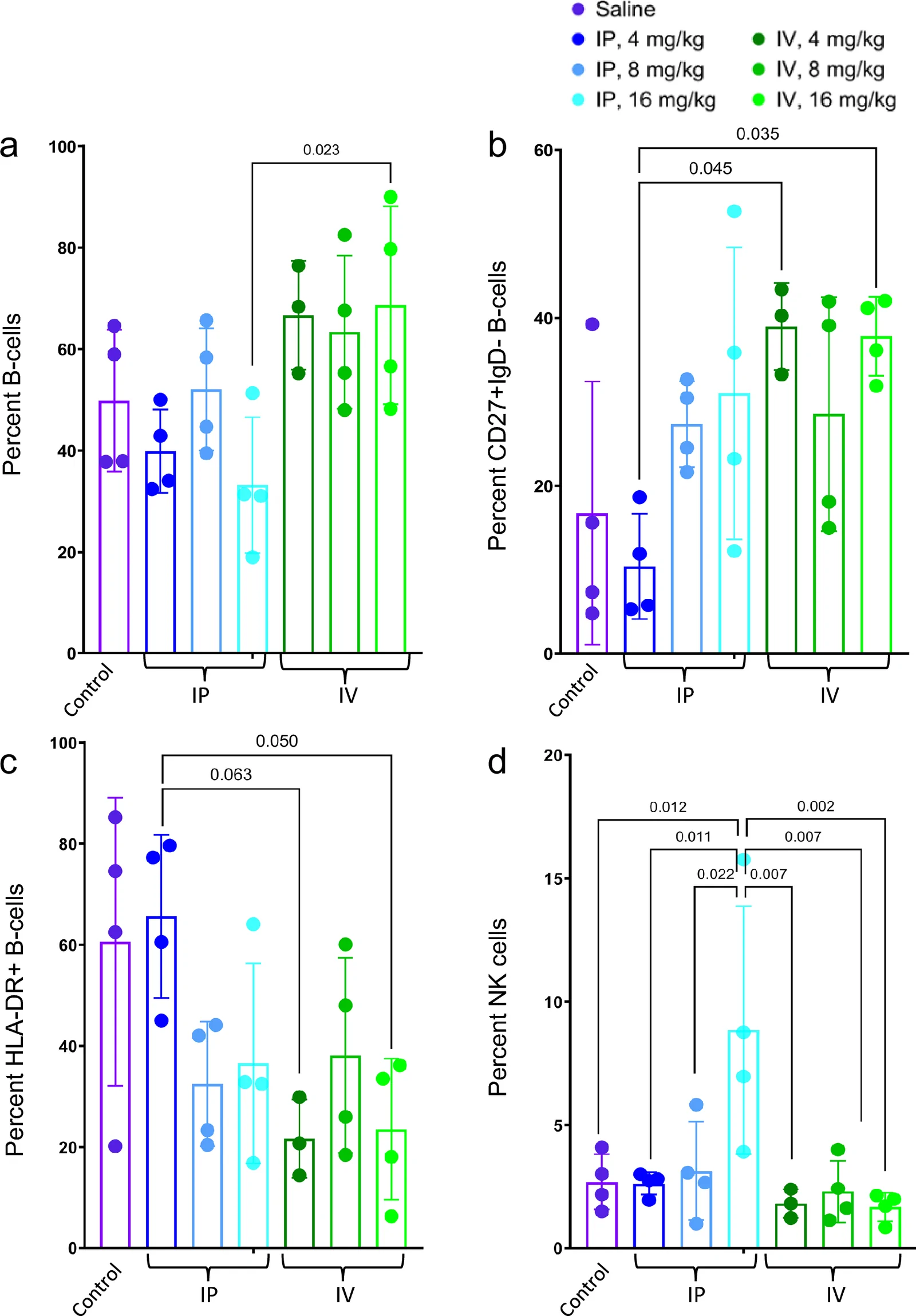
Human leukocyte subpopulations in blood at necropsy based on treatment. Shown are the percentage of B-cells (**a**) and percentage of NK cells (**d**) based on total human CD45 + cells. CD27 + IgD- B-cells (b) and HLA-DR + B-cells (**c**) are shown as a percentage of total B-cells. Ordinary one-way ANOVA with Tukey’s multiple comparison test was used for statistical analysis. Results shown as mean percent with standard deviation; statistically significant differences are *p* < 0.05

Total human whole blood leukocyte (WBL) cell counts showed no differences between any groups. T-cells and other subset populations (data not shown) had no differences in WBL while B-cell subsets (Fig. 3a–c) and NK cells (Fig. 3d) did show differences. B-cells (Fig. 3a) showed high-dose IP-treated different from high-dose IV-treated, percentage CD27 + IgD- (class-switched, Fig. 3b) had low-dose IP different from low- and high-dose IV, and HLA-DR + B-cells (Fig. 3c) low-dose IP were different from high-dose IV. NK cells (Fig. 3d) were significantly higher in high-dose IP-treated mice, compared to all other groups. The number of neutrophils per ul in WBL (Suppl Fig. 2a) was significantly decreased for donor C as compared to donor A, with *p* = 0.0536 versus control.

**Fig. 4:**
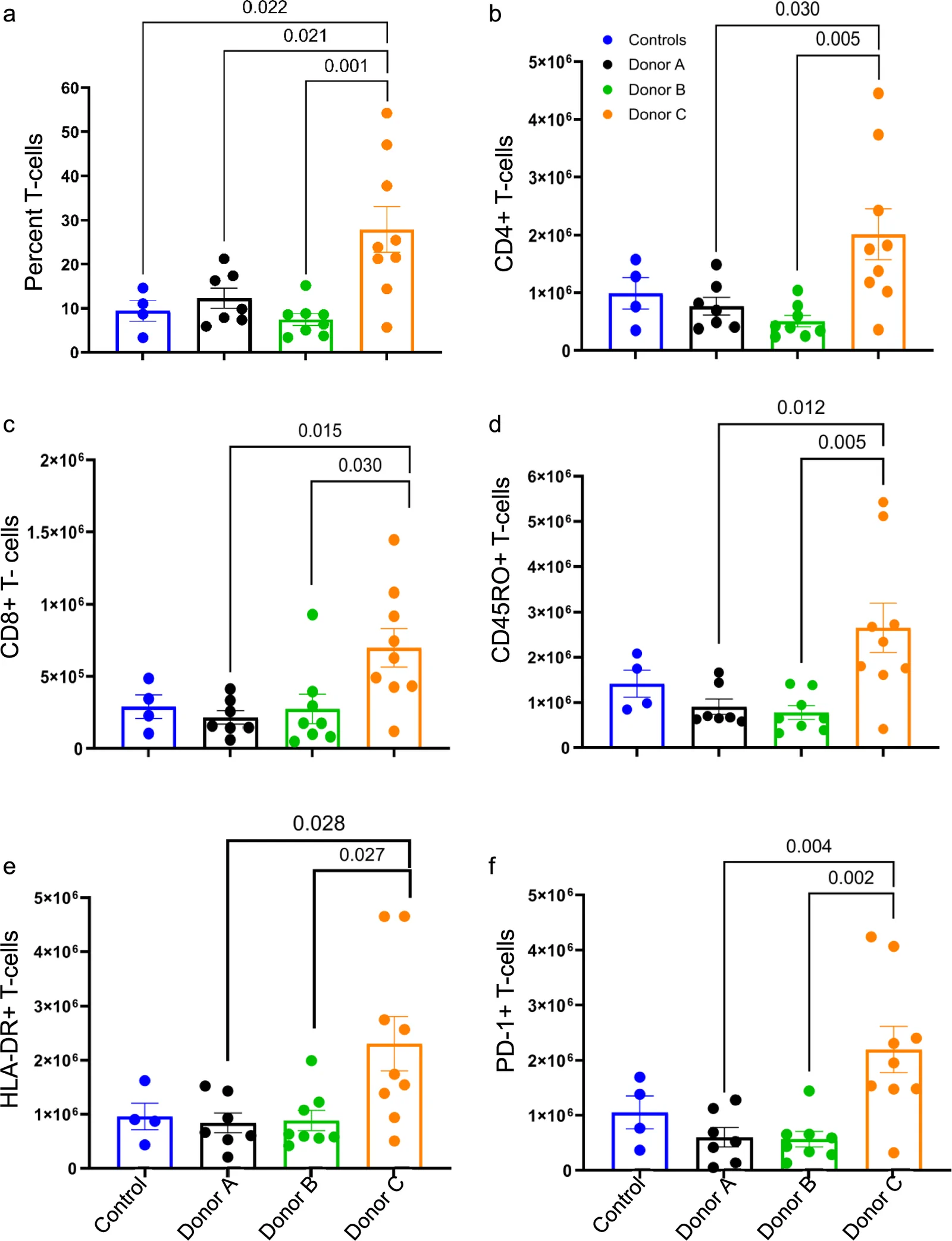
Human leukocytes in bone marrow based on donor. Cells were isolated, counted, and stained at necropsy. Shown are the percentage of total T-cells based on human CD45 + cells (**a**) and absolute cell counts for CD4 + T-cells (**b**), CD8 + T-cells (**c**), CD45RO + T-cells (**d**), HLA-DR + T-cells (**e**), and PD-1 + T-cells **(f**). Ordinary one-way ANOVA with Tukey’s multiple comparison test was used for statistical analysis. Results shown as mean percent (**a**) or mean total cells (**b**–**e**) with standard deviation; statistically significant differences are p < 0.05

No significant differences were observed in either bone marrow or spleen when comparing cell populations based on treatment (data not shown). However, in bone marrow, we observed an increased percentage of T-cells (Fig. 4a) for donor C, as compared to all other groups. The absolute number of CD4 + (Fig. 4b) and CD8 + (Fig. 4c) T-cells was significantly greater in donor C than in either donor A or B. We also noted increased activation of T-cells shown by absolute cell number increases for CD45RO + (Fig. 4d), HLA-DR + (Fig. 4e), and PD-1 + (Fig. 4f) T-cells for donor C as compared to both other donors. A trend of fewer neutrophils for donor C in bone marrow was noted, similar to what was observed in WBL (Suppl Fig. 2b).

Splenic cell populations showed similar trends as bone marrow when comparing these subpopulations based on donor; however, they were not significant (data not shown).

### HLA genetics

To investigate potential genetic factors underlying this donor-specific toxicity pattern, we examined the HLA profiles of all three donors (Table 1). The affected donor was homozygous for HLA-A*0201 and HLA-DPB1*0401. HLA-A*0201 is a class I allele, while HLA-DPB1*0401 is a class II allele. Class II alleles are involved in antigen presentation, where they bind to peptide fragments of proteins and display them on the cell surface for immune cells to recognize. The other donors were not homozygous at any alleles tested. There were no shared alleles for HLA-A between donors, but at least one allele for HLA-B, HLA-C, DPB1, DQB1, and DRB1 was shared with at least one other donor.

## Discussion

This pilot study was undertaken to determine an appropriate dose and route for copanlisib administration in BLT immune-humanized mice. In addition, we sought to determine if this animal model would develop adverse effects similar to those observed in clinical trials, and if so, at what dose level.

Clinical trials with copanlisib generally showed increased progression-free survival for a range of hematologic and solid tumors, but participants also experienced TEAE, which in some cases were grade 3–4 and included immune-mediated toxicities such as hepatitis, noninfectious colitis, and pneumonitis [7, 14, 15]. Other TEAE’s were skin toxicity, neutropenia, and opportunistic infections (1, 4, 12). In this study, we used three unique tissue donors to produce the immune-humanized mice and found that two of three had little to no findings at any dose, whereas the mice made from the third donor showed TRAEs at every dose, regardless of route. Histopathological analysis for that donor identified mild to moderate granulomas and/or focal lymphohistiocytic inflammation in lung, colon, pancreas, skin, bone marrow, and spleen. In addition, the colon of one treated mouse showed severe edema and hemorrhage with neutrophilic inflammation throughout. These findings are consistent with the immune-mediated toxicities observed in patients.

Other reports from clinical trials indicated that neutropenia was observed and that colonic biopsies showed T-cell infiltration [6, 7, 16–18]. The affected donor in this study had decreases in neutrophils in both PBMC and bone marrow, and flow cytometry showed bone marrow had increased number and activation level of T-cells, which could parallel T-cell infiltration observed in patients. Importantly, there was a control mouse in the saline group for the affected donor, and it had no pathology, supporting that these were TRAEs.

With respect to HLA typing, the genetic data suggest that HLA-A alleles could play a role in this toxicity. A study examining the correlation between irAEs and treatment outcome for CI therapy in nonsmall cell lung cancer identified that homozygous HLA-A*0201 patients had increased risk of irAEs, but improved response to therapy [19]. Another study in CI-treated patients found that homozygosity at more than one HLA-class I allele decreased risk of irAEs [20]. These studies and our pilot study results suggest that the adverse events observed in only one donor, even with sub-therapeutic levels of copanlisib, could be related to the individuals HLA haplotype.

Over the past two decades, emerging evidence has shown that immune-mediated adverse drug and hypersensitivity reactions have been linked to HLA haplotype for penicillin/*β*-lactam antibiotics, sulfonamides, antivirals, and antiseizure drugs [21–25]. Given the associations established for these drugs and emerging associations for CI therapy, it is possible that some TEAEs may have been specific to certain HLA haplotypes. While this would not account for all the adverse events experienced with copanlisib, it could explain why some trials had relatively few and/or mild TEAEs and other trials had much more serious AEs. It also suggests that routine HLA haplotype testing (including two fields, e.g., *02–01) might be important information to collect during a clinical trial, making correlation with AEs more straightforward. Products such as carbamazepine have a black box warning for HLA typing prior to commencing therapy to prevent those susceptible to AEs from taking the medication; this warning allows the drug to be administered to those who would not be expected to have adverse effects, while sparing affected individuals.

In summary, we show that PI3K adverse effects were present at each dosing level for one immune-humanized mouse donor, but not in any level for the other donors tested. This pilot study was limited by the use of only three donors and therefore cannot suggest HLA haplotype is a predictive biomarker. It does suggest that further research into the HLA haplotype of TEAEs associated with this drug class could help identify ‘at-risk’ patients, thereby aiding in the development of safe cancer therapeutics.

## Conclusion

No toxicity was observed in two of three donors regardless of dose or treatment route. For the single donor in which toxicity was observed, significant recruitment of activated T-cells correlated with inflammatory findings in lung, gastrointestinal tract, and bone marrow pathology. Although this finding only represents one donor from a group of three, it could suggest that individual immune responses might play a greater role in drug toxicity than has been fully investigated to date.

### Disclaimer

This article reflects the views of the author and should not be construed to represent FDA’s views or policies.

## Supplementary Information

Below is the link to the electronic supplementary material.

Supplementary file1 (PPTX 1614 KB)

Supplementary file2 (DOCX 26 KB)

Supplementary file3 (DOCX 27 KB)

Supplementary file4 (DOCX 28 KB)

Supplementary file5 (DOCX 59 KB)

## Data Availability

Data will be made available in accordance with U.S. FDA policy.
