# Supplementary material for: Donor-specific toxicity of copanlisib identified using immune-humanized mice
Source: Cancer Immunol Immunother. 2026 Jun 12;75(9):212. doi: 10.1007/s00262-026-04398-9 (PMC13490038; doi:10.1007/s00262-026-04398-9)
Supplement: Supplementary file 1 [file 262_2026_4398_MOESM2_ESM.docx]

**Supplemental table 1a.** Summary of number of mice by treatment and study endpoint.

| Treatment Group | WBL  D0 | WBL D14 | WBL NEC | SP/BM NEC | Pathology |  |
| --- | --- | --- | --- | --- | --- | --- |
| IP, 4 mg/kg | 4 | 4 | 4 | 4 | 4 |  |
| IP, 8 mg/kg | 4 | 4 | 4 | 4 | 4 |  |
| IP, 16 mg/kg | 4 | 4 | 4 | 4 | 4 |  |
|  |  |  |  |  |  |  |
| IV, 4 mg/kg | 4 | 4 | 4* | 4* | 4* |  |
| IV, 8 mg/kg | 4 | 3 | 4* | 4* | 4* |  |
| IV, 16 mg/kg | 4 | 4 | 4 | 4 | 4 |  |
|  |  |  |  |  |  |  |
| Saline - IP | 4 | 4 | 4 | 4 | 4 |  |

* - includes interim necropsy data

**Supplemental table 1b.** Summary of number of mice by donor and study endpoint.

| Donor Group | WBL  D0 | WBL D14 | WBL NEC | SP/BM NEC | Pathology |  |
| --- | --- | --- | --- | --- | --- | --- |
| Donor A | 7 | 6 | 7* | 7* | 7* |  |
| Donor B | 8 | 8 | 8* | 8* | 8* |  |
| Donor C | 9 | 9 | 9 | 9 | 9 |  |
| Saline - IP | 4 | 4 | 4 | 4 | 4 |  |

* - includes interim necropsy data
