# Supplementary material for: Donor-specific toxicity of copanlisib identified using immune-humanized mice
Source: Cancer Immunol Immunother. 2026 Jun 12;75(9):212. doi: 10.1007/s00262-026-04398-9 (PMC13490038; doi:10.1007/s00262-026-04398-9)
Supplement: Supplementary file 2 [file 262_2026_4398_MOESM3_ESM.docx]

**Supplemental table 2.** Individual study mice shown by treatment route, with donor noted.

| Group # | Study | Route | Dose | D0 | D14 | NEC |
| --- | --- | --- | --- | --- | --- | --- |
| Donor A | Copanlisib | IP | 4 mg/kg | 1 | 1 | 1 |
| Donor B | Copanlisib | IP | 4 mg/kg | 1 | 1 | 1 |
| Donor C | Copanlisib | IP | 4 mg/kg | 1 | 1 | 1 |
| Donor C | Copanlisib | IP | 4 mg/kg | 1 | 1 | 1 |
|  |  |  |  |  |  |  |
| Donor A | Copanlisib | IP | 8 mg/kg | 1 | 1 | 1 |
| Donor B | Copanlisib | IP | 8 mg/kg | 1 | 1 | 1 |
| Donor C | Copanlisib | IP | 8 mg/kg | 1 | 1 | 1 |
| Donor C | Copanlisib | IP | 8 mg/kg | 1 | 1 | 1 |
|  |  |  |  |  |  |  |
| Donor A | Copanlisib | IP | 16 mg/kg | 1 | 1 | 1 |
| Donor B | Copanlisib | IP | 16 mg/kg | 1 | 1 | 1 |
| Donor C | Copanlisib | IP | 16 mg/kg | 1 | 1 | 1 |
| Donor C | Copanlisib | IP | 16 mg/kg | 1 | 1 | 1 |
|  |  |  |  |  |  |  |
| Donor A | Copanlisib | IV | 4 mg/kg | 1 | 1 | 1 |
| Donor B | Copanlisib | IV | 4 mg/kg | 1 | 1 | 1 |
| Donor B | Copanlisib | IV | 4 mg/kg | 1 | 1 | 0/1 |
| Donor C | Copanlisib | IV | 4 mg/kg | 1 | 1 | 1 |
|  |  |  |  |  |  |  |
| Donor A | Copanlisib | IV | 8 mg/kg | 1 | 0 | 0/1 |
| Donor B | Copanlisib | IV | 8 mg/kg | 1 | 1 | 1 |
| Donor B | Copanlisib | IV | 8 mg/kg | 1 | 1 | 1 |
| Donor C | Copanlisib | IV | 8 mg/kg | 1 | 1 | 1 |
|  |  |  |  |  |  |  |
| Donor A | Copanlisib | IV | 16 mg/kg | 1 | 1 | 1 |
| Donor A | Copanlisib | IV | 16 mg/kg | 1 | 1 | 1 |
| Donor B | Copanlisib | IV | 16 mg/kg | 1 | 1 | 1 |
| Donor C | Copanlisib | IV | 16 mg/kg | 1 | 1 | 1 |
|  |  |  |  |  |  |  |
| Donor A | Copanlisib | IP | Saline | 1 | 1 | 1 |
| Donor A | Copanlisib | IP | Saline | 1 | 1 | 1 |
| Donor B | Copanlisib | IP | Saline | 1 | 1 | 1 |
| Donor C | Copanlisib | IP | Saline | 1 | 1 | 1 |
