# Supplementary material for: Donor-specific toxicity of copanlisib identified using immune-humanized mice
Source: Cancer Immunol Immunother. 2026 Jun 12;75(9):212. doi: 10.1007/s00262-026-04398-9 (PMC13490038; doi:10.1007/s00262-026-04398-9)
Supplement: Supplementary file 4 [file 262_2026_4398_MOESM5_ESM.docx]

Table 1. Summary of Results from Histopathological Analyses of Copanlisib Treated Immune-Humanized Mice

Donor A	Donor B	Donor C________________Saline___

IP	IV	IP	IV	IP	IV	IP

Mouse number: IP: 4, 8 or 16mg/kg	2041 2042	2035	2039	2109	2125	2101	2122 2110	2137	2150 2126	2145	2148	2040 2045

IV: 4, 8 or 16mg/kg	2043	2034	2037	2103	2115	2116	2128	2142	2149	2134	2124 2132

| Spleen |  |  |  |  |  |  |  |  |  |  |  |  |  |  |  |  |  |  |  |  |
| --- | --- | --- | --- | --- | --- | --- | --- | --- | --- | --- | --- | --- | --- | --- | --- | --- | --- | --- | --- | --- |
| Extramedullary hematopoiesis | **++**  **+** | **+++** | **++**  **+++** | **+**  **+++** |  | ++  ++ | **++** | **++**  **++** | **++**  **++** | **+++** |  | **++**  **++** | **++**  **++** | **+**  **+** | **++**  **++** | **+++** |  | **++**  **++** | **++**  **+** |  |
| LH inflammation | **-**  **-** | **-** | **-**  **-** | **-**  **-** |  | -  - | **-** | **-**  **-** | **-**  **-** | **-** |  | **-**  **-** | **-**  **-** | **-**  **-** | **-**  **-** | **-** |  | **-**  **-** | **-**  **-** |  |
| Granulomas | **-**  **-** | **-** | **-**  **-** | **+**  **-** |  | -  - | **-** | **-**  **-** | **+**  **-** | **-** |  | **++**  **-** | **-**  **++** | **+**  **++** | **++**  **++** | **-** |  | **-**  **-** | **-**  **-** |  |
| Endocrine organs |  |  |  |  |  |  |  |  |  |  |  |  |  |  |  |  |  |  |  |  |
| LH pancreatitis | **+**  **-** | **-** | **-**  **-** | **-**  **-** |  | -  - | **+** | **-**  **-** | **-**  **-** | **-** |  | **+**  **-** | **-**  **-** | **-**  **-** | **++**  **-** | **-** |  | **-**  **-** | **+**  **-** |  |
| Pituitary granulomas | **-**  **-** | **-** | **-**  **-** | **-**  **-** |  | -  - | **-** | **-**  **-** | **-**  **-** | **-** |  | **-**  **-** | **-**  **-** | **-**  **-** | **+++**  **+** | **-** |  | **-**  **-** | **-**  **-** |  |
| Pituitary amyloid deposits | **-**  **-** | **-** | **-**  **+** | **+**  **-** |  | -  - | **-** | **+**  **+** | **-**  **-** | **+** |  | **-**  **-** | **-**  **-** | **-**  **-** | **+**  **-** | **-** |  | **+**  **-** | **-**  **+** |  |
| Musculoskeletal System |  |  |  |  |  |  |  |  |  |  |  |  |  |  |  |  |  |  |  |  |
| LH myositis (sternum and/or femur) | **-**  **-** | **-** | **-**  **-** | **-**  **-** |  | **-**  **-** | **-** | **-**  **-** | **-**  **-** | **-** |  | **++**  **+** | **+**  **++** | **++**  **++** | **-**  **++** | **-** |  | **-**  **-** | **-**  **-** |  |
| LH osteomyelitis (granulomas) | **++**  **-** | **-** | **-**  **-** | **-**  **+** |  | **-**  **-** | **-** | **-**  **-** | **-**  **-** | **-** |  | **++**  **+** | **+**  **++** | **++**  **++** | **+++**  **++** | **-** |  | **-**  **-** | **+**  **-** |  |
| LH synovitis | **-**  **-** | **-** | **-**  **-** | **-**  **-** |  | **-**  **-** | **-** | **-**  **-** | **-**  **-** | **-** |  | **-**  **-** | **-**  **-** | **-**  **-** | **-**  **-** | **-** |  | **-**  **-** | **-**  **-** |  |
|  |  |  |  |  |  |  |  |  |  |  |  |  |  |  |  |  |  |  |  |  |
| Skin |  |  |  |  |  |  |  |  |  |  |  |  |  |  |  |  |  |  |  |  |
| Epidermal hyperplasia and hyperkeratosis | **-**  **-** | **-** | **-**  **-** | **-**  **-** |  | **-**  **-** | **-** | **-**  **-** | **-**  **-** | **-** |  | **-**  **-** | **-**  **-** | **-**  **-** | **-**  **-** | **-** |  | **-**  **-** | **-**  **-** |  |
| Epidermal or subepidermal LH infiltration | **-**  **-** | **-** | **-**  **-** | **-**  **-** |  | **-**  **-** | **-** | **-**  **-** | **-**  **-** | **-** |  | **+**  **-** | **-**  **+** | **+**  **-** | **+**  **+** | **-** |  | **-**  **-** | **-**  **-** |  |
| Dermal LH cellular infiltration (dermatitis) | **+**  **-** | **-** | **+**  **-** | **-**  **-** |  | **-**  **-** | **-** | **-**  **-** | **-**  **-** | **-** |  | **-**  **-** | **-**  **-** | **-**  **-** | **-**  **-** | **-** |  | **-**  **-** | **-**  **-** |  |
|  |  |  |  |  |  |  |  |  |  |  |  |  |  |  |  |  |  |  |  |  |
| Gastrointestinal tract |  |  |  |  |  |  |  |  |  |  |  |  |  |  |  |  |  |  |  |  |
| LH or neutrophilic inflammation | **+**  **-** | **-** | **-**  **-** | **-**  **-** |  | **-**  **-** | **-** | **-**  **-** | **-**  **-** | **-** |  | **+**  **++** | **+**  **-** | **+**  **++** | **-**  **-** | **+** |  | **-**  **-** | **-**  **-** |  |
| Amyloid deposits | **+**  **+** | **-** | **-**  **-** | **+**  **-** |  | **+**  **-** | **+** | **-**  **-** | **+**  **-** | **-** |  | **-**  **-** | **-**  **-** | **-**  **-** | **-**  **-** | **-** |  | **+**  **+** | **-**  **-** |  |

Summary of Results from Histopathological Analyses of Copanlisib Treated Immune-Humanized Mice

Donor A	Donor B	Donor C___________	Saline

IP	IV	IP	IV	IP	IV	IP

Mouse number: IP: 4, 8 or 16mg/kg	2041	2042	2035	2039	2109	2125	2101	2122	2110	2137	2150 2126	2145	2148	2040 2045

IV: 4, 8 or 16mg/kg	2043	2034	2037	2103	2115	2116	2128	2142	2149	2134	2124 2132

| Lung |  |  |  |  |  |  | |  | |  | |  | |  |  | |  | |  | |  |  | |  |  |  |  |
| --- | --- | --- | --- | --- | --- | --- | --- | --- | --- | --- | --- | --- | --- | --- | --- | --- | --- | --- | --- | --- | --- | --- | --- | --- | --- | --- | --- |
| Peribronchiolar and perivascular LH | -  - | - | -  + | -  - |  | -  - | - | | -  - | | -  - | | - | |  | -  - | | -  - | | -  + | | +  - | + | |  | +  - | -  - |

| Infiltration | +  - | - | -  - | -  - |  | -  - | - | -  - | -  - | - |  | -  - | -  - | -  - | -  - | - |  | -  - | -  - |
| --- | --- | --- | --- | --- | --- | --- | --- | --- | --- | --- | --- | --- | --- | --- | --- | --- | --- | --- | --- |
| Granuloma | -  - | - | -  - | -  - |  | -  - | - | -  - | -  - | - |  | -  - | -  - | +  - | -  - | - |  | -  - | -  - |
|  |  |  |  |  |  |  |  |  |  |  |  |  |  |  |  |  |  |  |  |
| Liver |  |  |  |  |  |  |  |  |  |  |  |  |  |  |  |  |  |  |  |
| Extramedullary hematopoiesis | -  - | - | -  - | -  - |  | -  - | - | -  - | -  - | - |  | -  - | -  - | -  - | -  - | + |  | -  - | -  - |
| Parenchymal granuloma | -  - | - | -  - | +  - |  | -  - | - | -  - | -  - | - |  | -  - | -  - | -  - | -  - | - |  | -  - | -  - |
| Necrosis | -  - | - | -  - | +  - |  | -  - | - | -  - | -  - | - |  | -  - | -  - | -  - | -  - | - |  | -  - | -  - |
|  |  |  |  |  |  |  |  |  |  |  |  |  |  |  |  |  |  |  |  |

Abbreviations: LH – lympho-histiocytic.

Note: Amyloid deposits were primarily found in the intestine and pituitary gland in mice from all groups. We have observed this in other studies, and it does not appear to be a test article effect, but is rather associated with the humanized mouse model.
